# Experiences of patients undergoing IVF treatment during the COVID-19 pandemic

**DOI:** 10.5935/1518-0557.20230002

**Published:** 2023

**Authors:** Marwa Elfatih Fadailu, Solaiman Al Obaid, Ouhoud Kaddour, Fatima AlQahtani, Dania Al-Jaroudi

**Affiliations:** 1 Obstetrics and Gynecology Department, Al-Yamama Maternity and Children Hospital, Riyadh, Saudi Arabia; 2 Obstetrics, and Gynecology and Reproductive Endocrine & Infertility Medicine Department. Women’s Specialized Hospital-KFMC, Riyadh, Saudi Arabia; 3 Medical Researches Company (MRC), Riyadh, Saudi Arabia

**Keywords:** COVID-19, *in vitro* fertilization, infertility treatment suspension, infertile patient’s response

## Abstract

**Objective:**

To assess the willingness of patients with infertility to continue with their in vitro fertilization (IVF) treatment during the COVID-19 pandemic.

**Methods:**

This cross-sectional survey was conducted in the reproductive, endocrine, and infertility medicine department (REIMD) at King Fahad Medical City (KFMC), Riyadh, Saudi Arabia. Patients that were planned to undergo IVF treatment at REIMD were contacted and asked about whether they would like to start IVF treatment during the COVID-19 pandemic from August 2020 to August 2021. Data was analyzed using the SPSS version 24. Statistics obtained as means and standard deviations from continuous variables correlated with the Chi-square test and results were considered significant at *p*≤0.05.

**Results:**

Of the 400 participants, 245 (61.25%) were between the ages of 30-39 years. About 42.75% (n=171) of the patients had 6-10 years of infertility, and 18% (n=72) had at least one pregnancy but no living children. While 64.7% (n=259) of the participants responded on the first call, 83% (n=332) agreed to continue their treatment. Of those, 13% (n=43) preferred to book appointments as soon as possible; 29.8% (n=99) preferred booking within three months; while 57.2% (n=190) chose to book after three months. From our sample, 86.8% (n=59) were afraid to contract the virus and the choice to delay the IVF treatment correlated with the patient’s age (*p*<0.001) and duration of infertility (*p*=0.007).

**Conclusions:**

The COVID-19 pandemic affected IVF treatment courses, and many patients were afraid to be infected during this pandemic.

## INTRODUCTION

In December 2019, the Hubei Province in China alerted the world about a possible outbreak of a type of pneumonia of unknown etiology at the time, later on named SARS-COV-2 or COVID-19 (Saksena *et al*., 2021). This novel virus was found to be highly infectious with high mortality rates and was consequently labeled as a pandemic by the World Health Organization (WHO) (Hanaei & Rezaei, 2020).

The COVID-19 pandemic represents a challenge for the global healthcare community; therefore, clinical and public health guidance made efforts to decrease its spread using different strategies including recommendations to limit healthcare provision to critical and emergency cases only and delay selective care (La Marca *et al*., 2020).

Since infertility is not classified as a serious medical condition, all procedures related to its treatment were temporarily postponed to reduce the risk of exposure to COVID-19 (Tokgoz *et al*., 2022; Rodriguez-Wallberg & Wikander, 2020; Bhattacharya *et al*., 2021). The patients reported high levels of anxiety and agitation due to treatment suspension (Tokgoz *et al*., 2022).

Some assisted reproduction and infertility specialists opposed postponing infertility treatments during this pandemic, as some patients require urgent fertility preservation procedures (Tokgoz *et al*., 2022; Romanski *et al*., 2020).

When infertility treatment was suspended during the surge of the COVID-19 pandemic, patients felt helpless, and most of them wanted to continue their treatment, which was associated with a higher rate of distress (Ben-Kimhy *et al*., 2020). Upon assessing the psychological impact of the COVID-19 pandemic on infertility patients, coronavirus was the third most common stressor among respondents in the early phase of the COVID-19 pandemic (Vaughan *et al*., 2020; Robertson *et al*., 2021).

Recently, the reduction of patient contact in healthcare facilities enabled a safe access for patients to continue their infertility treatments.

In Saudi Arabia, the effects of COVID-19 on the treatment of infertility have not been well studied. This study dwells into the latter and offers one of the first evidence with this focus that can help shed light and build towards larger studies.

## MATERIALS AND METHODS

### Study design

A cross-sectional survey included 400 subfertile Saudi females who planned for an assisted reproduction treatment cycle that was suspended due to the COVID-19 pandemic. Data was collected from August 2020 to August 2021.

### Study settings

Reproductive, endocrine, and infertility medicine department (REIMD) at King Fahad Medical City (KFMC), Riyadh, Saudi Arabia.

### Study Population

A total of 400 Saudi subfertile females were contacted by phone from those registered at the REIMD for *in vitro* fertilization (IVF) treatment to assess their response to the commencement of their IVF treatment post-lockdown and quarantine.

### Eligibility Criteria

Patients who responded to phone calls and had been on the waiting list for the commencement of IVF treatment and responded were included in this study.

### Study Outcome

Willingness of patients suffering from infertility on continuing with their IVF treatment during the COVID-19 pandemic and affected factors.

### Data collection tools

The validated questionnaire included questions about patient demographics, medical history including previous pregnancies and duration of infertility. Additionally, we recorded data on the patient’s willingness and attitude to continue with IVF treatment and attitude towards.

### Ethical consideration

The institutional review board (IRB) approval was obtained under the number 21-100.

### Statistical methods

The analysis was performed using the SPSS version 24. The results were obtained as means, medians, standard deviations from continuous variables, and frequency and percentages for categorical and qualitative variables. Univariate tests of association between qualitative variables were undertaken using Chi-square/Fisher’s Exact tests. Tests of significant differences in quantitative/continuous variables between or among groups were conducted based on the t-test/nonparametric methods and analysis of variance techniques. An overall 5% significance was used for all analyses.

## RESULTS

A total of 400 Saudi infertile females responded to the call and were contacted using their personal information available in their hospital files.

More than half of the participants were 30-39 years of age - 61.25% (n=245); while 14% (n=56) of the patients were above 39 years of age. Occupation status of the participants revealed that a majority of them were housewives 85.25% (n=341). Regarding infertility duration, almost half - 42.75% (n=171) of the females reported the duration of infertility to be 6-10 years. When questioned regarding their previous pregnancy, 18% (n=72) responded they were pregnant once before. With regard to the booking status, more than half 83% (n=332) reported to have rebooked their appointments (Table 1).

**Table 1:** Characteristics of the study population (n=400).

Characteristics		Results
**Age (years)**	Mean±SD	33.19±5.07
	20-29 years	24.75% (99/400
	30-39 years	61.25% (245/400)
	40-49 years	14.00% (56/400)
**Occupation**	Housewife	85.25% (341/400)
	Employee	9.75% (39/400)
	Teacher	2.75% (11/400)
	Nurse	1.50% (6/400)
	Head of office	0.25% (1/400)
	Ministry of health	0.25% (1/400)
	Dental assistant	0.25% (1/400)
**Duration of Infertility (years)**	Mean	7.09±3.77
	1-5 years	40.50% (162/400)
	6-10 years	42.75% (171/400)
	11-15 years	13.50% (54/400)
	16-20 years	2.5% (10/400)
	> 20 years	0.75% (3/400)
**previous pregnancy**	Previous Pregnancy	18% (72/400)
	No Previous Pregnancy	82% (328/400)
**previous delivery**	Previous Delivery	01% (4/400)
	No Previous Delivery	99% (396/400)
**Booking status**	Rebooked	83.00% (332/400)
	Not booked	17/00 (68/400)

Table 2 shows the details of the patients’ responses to their treatment plans. Regarding the response to hospital calls, 259 patients got the call one time (64.7%); 127 (31.8%) patients got the call two times; 8 (2%) patients got the call three times; and 6 (1.5%) patients got the call four times. The patients who agreed to continue their *in vitro* fertilization (IVF) treatment made up a majority 83% (n=332). With regards to the preferred date, more than half of the respondents, 190 (57.2%), reported to receive an appointment after 3 months. Fear of acquiring COVID-19 infection was revealed to be the most common reason to reschedule treatment at a later date, with 86.8% (n=59) of the respondents reporting it. The least common reason to discontinue treatment was pregnancy, with only 1.5% (n=1) reporting it.

**Table 2:** Details of the patient response to be scheduled for their management plan.

		Number	%
**Pt. Response Agreement status**	Agreed	332	83.0
	Did not agree	68	17.0
**Pt. Response: Agreed**	ASAP	43	13.0
	Within 3 Months	99	29.8
	After 3 Months	190	57.2
**Pt. Response: Did NOT Agree**	Spontaneous pregnancy	1	1.5
	Fear of acquiring infection	59	86.8
	Positive COVID-19	5	7.4
	Following Somewhere else	3	4.4

There was a statistically significant association between preferred date and age (*p*<0.001), as well as with the duration of infertility (*p*=0.007) (Table 3). There was no significant association between occupation and preferred date.

**Table 3:** Distribution according to the treatment continuation and correlation with preference.

		ASAP (n=43)		Within 3 Months (n=99)		After 3 Months (n=190)		*p* value
		Number	%	Number	%	Number	%	
**Age**	20-29 years	6	13.95	13	13.13	70	36.84	<0.001*
	30-39 years	21	48.84	66	66.67	113	59.47	
	40-49 years	16	37.21	20	20.20	7	3.68	
**Occupation**	Housewife	35	81.40	85	85.86	166	87.37	0.516
	Employee	4	9.30	10	10.10	19	10.00	
	Teacher	2	4.65	1	1.01	3	1.58	
	other	2	4.65	3	3.03	2	1.05	
**Duration of Infertility**	1-5 years	13	30.23	35	35.35	87	45.79	0.007*
	6-10 years	17	39.53	42	42.42	85	44.74	
	11-15 years	11	25.58	19	19.19	13	6.84	
	16-20 years	2	4.65	1	1.01	4	2.11	
	> 20 years	0	0.00	2	2.02	1	0.53	

* Significant *p* value.

## DISCUSSION

This study explored the effects of the COVID-19 pandemic on infertility patients who were forced to suspend their treatments during the pandemic. Most patients preferred to resume treatment despite possible risks and uncertainties. The remaining patients were afraid of acquiring the infection if they opted to continue with the treatment.

In this study, the patients who agreed to resume their *in vitro* fertilization (IVF) treatment were the majority. In contrast, only 1 out of 6 (17%) patients delayed treatment due to the COVID-19 pandemic.

This study describes that fear of infection was related to the suspension of fertility treatments due to the COVID-19 pandemic. Despite the risk of COVID-19 infection, most of the patients, during the peak of the pandemic, believed the decision to suspend treatments by the authorities was unjustified, and most of them would have chosen to resume fertility treatments if given a choice (Bhattacharya *et al*., 2021; Marom Haham *et al*., 2021). Patients suffering from infertility, being mostly less than 40 years of age and healthy, are not a risk group for COVID-19 complications (Tokgoz *et al*., 2022; Rodriguez-Wallberg & Wikander, 2020; Ben-Kimhy *et al*., 2020). This might explain why most patients would have liked to resume treatment despite the risk of being exposed to COVID-19, in line with the health belief model, suggesting that lower perceptions of susceptibility justify a lower perceived need for prevention (Rosenstock *et al*., 1988). The willingness to resume treatment despite the current situation, was not associated with background characteristics (Turocy *et al*., 2020; Gleicher, 2020; Robertson *et al*., 2021). It seems that since all these patients made a decision before the COVID-19 pandemic to undergo fertility treatment despite the difficulties accompanying this process, they all, regardless of personal and social differences, wished to continue pursuing their treatment. Patients undergoing fertility treatments, at this time, in addition to the general distress due to the pandemic, face a huge emotional burden of cycle cancellation for an indefinite period of time.

This study finding indicates the women’s willingness to continue treatment was associated with their age. Being at younger age and long duration of infertility were related factors for suspension of treatments; in contrast to studies which correlated this with their background (Rodriguez-Wallberg & Wikander, 2020; Bhattacharya *et al*., 2021; Smith *et al*., 2020; Gordon & Balsom, 2020).

Caregivers may use the data generated in this study to reduce the stress and the psychological effect of our infertility patients during the COVID-19 pandemic.

The study had several limitations, including the single-center nature of the participants included, the respondent bias as the questions were orally given during the phone calls and the participants’ answers could be affected by the presence of the researcher. A larger study with multi-center participants is advised for a better understanding of this population, and to develop protocols to help better care for them.

## CONCLUSION

The COVID-19 pandemic markedly affected IVF treatment cycles, and many patients were afraid of getting an infection during their IVF procedures. However, most patients preferred to resume treatment despite possible risks of infection and uncertainties. A multicenter study with a larger sample is recommended to better understand the long term indirect effects that the pandemic has had on the management and IVF treatment of people suffering from infertility.
